# 1,5-Bis[(*E*)-1-(2-hydroxyphenyl)ethyl­idene]thiocarbonohydrazide mono­hydrate

**DOI:** 10.1107/S1600536810004241

**Published:** 2010-02-06

**Authors:** Md. Abu Affan, Dayang N. A. Chee, Fasihuddin B. Ahmad, Edward R. T. Tiekink

**Affiliations:** a Department of Chemistry, Faculty of Resource Science and Technology, Universiti Malaysia Sarawak, 94300 Kota Samarahan, Sarawak, Malaysia; b Department of Chemistry, University of Malaya, 50603 Kuala Lumpur, Malaysia

## Abstract

In the title compound, C_17_H_18_N_4_O_2_S·H_2_O, the thio­urea derivative is almost planar, with an r.m.s. deviation for the non-H atoms of 0.057 Å. The hydroxyl groups lie to the same side of the mol­ecule as the thione S atom, a conformation that allows the formation of intra­molecular O—H⋯S and O—H⋯N hydrogen bonds. In the crystal structure, the thio­urea and water mol­ecules self-assemble into a two-dimensional array by a combination of O_water_—H⋯O_hydrox­yl_, N—H⋯O_water_ and O_water_—H⋯S hydrogen bonds and C—H⋯π inter­actions.

## Related literature

For background and recent studies of the biological activity of organotin compounds, see: Gielen & Tiekink (2005 ▶); Affan *et al.* (2009 ▶). For the structure of the ketone analogue of the title compound, see: Zukerman-Schpector *et al.* (2009 ▶).
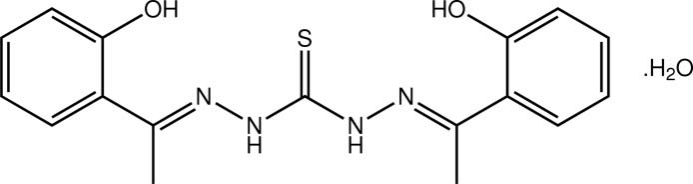


## Experimental

### 

#### Crystal data

C_17_H_18_N_4_O_2_S·H_2_O
*M* *_r_* = 360.43
Monoclinic, 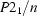

*a* = 15.8654 (3) Å
*b* = 7.3938 (1) Å
*c* = 16.3697 (3) Å
β = 115.922 (1)°
*V* = 1727.06 (5) Å^3^
*Z* = 4
Mo *K*α radiation
μ = 0.21 mm^−1^
*T* = 100 K
0.44 × 0.13 × 0.07 mm

#### Data collection

Bruker SMART APEXII CCD diffractometer
Absorption correction: multi-scan (*SADABS*; Sheldrick, 1996 ▶) *T*_min_ = 0.905, *T*_max_ = 1
15464 measured reflections
3968 independent reflections
3136 reflections with *I* > 2σ(*I*)
*R*_int_ = 0.033

#### Refinement

*R*[*F* ^2^ > 2σ(*F* ^2^)] = 0.038
*wR*(*F* ^2^) = 0.102
*S* = 1.03
3968 reflections
246 parameters
7 restraints
H atoms treated by a mixture of independent and constrained refinement
Δρ_max_ = 0.35 e Å^−3^
Δρ_min_ = −0.27 e Å^−3^

### 

Data collection: *APEX2* (Bruker, 2007 ▶); cell refinement: *SAINT* (Bruker, 2007 ▶); data reduction: *SAINT*; program(s) used to solve structure: *SHELXS86* (Sheldrick, 2008 ▶); program(s) used to refine structure: *SHELXL97* (Sheldrick, 2008 ▶); molecular graphics: *ORTEP-3* (Farrugia, 1997 ▶) and *DIAMOND* (Brandenburg, 2006 ▶); software used to prepare material for publication: *SHELXL97*.

## Supplementary Material

Crystal structure: contains datablocks global, I. DOI: 10.1107/S1600536810004241/hb5327sup1.cif

Structure factors: contains datablocks I. DOI: 10.1107/S1600536810004241/hb5327Isup2.hkl

Additional supplementary materials:  crystallographic information; 3D view; checkCIF report

## Figures and Tables

**Table 1:** Hydrogen-bond geometry (Å, °) *Cg*1 is the centroid of the C4–C9 ring.

*D*—H⋯*A*	*D*—H	H⋯*A*	*D*⋯*A*	*D*—H⋯*A*
O1—H1*O*⋯N2	0.83 (2)	1.79 (2)	2.5191 (17)	147 (2)
O1—H1*O*⋯S1	0.83 (2)	2.86 (2)	3.5126 (13)	138 (2)
O2—H2*O*⋯N4	0.82 (2)	1.81 (2)	2.542 (2)	148 (2)
O2—H2*O*⋯S1	0.82 (2)	2.96 (2)	3.6220 (15)	139 (2)
O3—H3*O*⋯O1	0.84 (2)	1.91 (2)	2.7525 (18)	174 (2)
O3—H4*O*⋯S1^i^	0.82 (2)	2.76 (2)	3.5089 (14)	154 (2)
N1—H1*N*⋯O3^ii^	0.87 (2)	2.04 (2)	2.8169 (19)	150 (2)
N3—H3*N*⋯O3^ii^	0.88 (2)	2.04 (2)	2.854 (2)	154 (2)
C11—H11*C*⋯*Cg*1^iii^	0.98	2.61	3.4497 (17)	144

Symmetry codes: (i) 
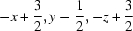
; (ii) 
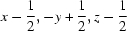
; (iii) 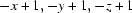.

**Fig. 1.**
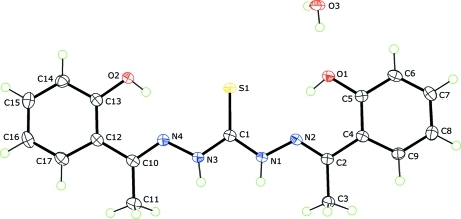
Molecular structure of (I) showing displacement ellipsoids at the 50% probability level.

**Fig. 2.**
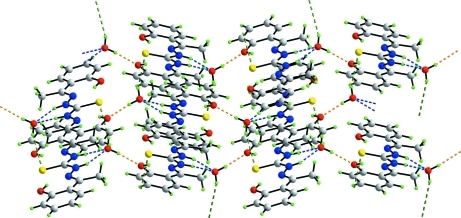
Supramolecular 2-D array in (I) mediated by O—H···O (orange dashed lines), N—H···O (blue dashed lines) and O—H···S (green dashed lines) hydrogen bonds. Colour code: S, yellow; O, red; N, blue; C, grey; H, green.

## supplementary crystallographic information

### Comment

The molecule in the title hydrate, (I), was synthesised as part of an on-going study into biological studies of organotin compounds (Gielen & Tiekink, 2005; Affan *et al.*, 2009). The thiourea derivative in (I) is effectively planar with the maximum deviation of any of the torsion angles from 0 or 180° being 4.6 (2)° for N4—C10—C12—C13 and -174.62 (15)° for C11—C10—C12—C13. The r.m.s. deviation for all non-hydrogen atoms in the thiourea molecule = 0.057 Å. The conformation about each of the C1═N2 [1.290 (2) Å] and C10═N4 [1.2956 (19) Å] double bonds is *E*. Finally, the hydroxyl groups are orientated to the same side of the molecule as the thione-S atom. The described molecular conformation is stabilised by intramolecular O_hydroxyl_—H···N_imine_ and O_hydroxyl_—H···S_thione_ hydrogen bonds, Table 1. Allowing for substitution of the thione by a ketone group, the described molecular conformation for the thiourea molecule in (I) resembles that in the recently reported ketone derivative (Zukerman-Schpector *et al.*, 2009).

In addition to the intramolecular hydrogen bonds, the crystal structure features O_water_—H···O_hydroxyl_, N—H···O_water_ and O_water_—H···S hydrogen bonds, Table 1. The N—H atoms of one molecule connect to a water molecule which in turn forms a hydrogen bond with a hydroxyl-O1 atom of a second thiourea derivative. These interactions give rise to a supramolecular chain with base vector [101]. However, this does not take into account a second donor hydrogen bond involving the water molecule. This forms a hydrogen bond with a thione-S atom of a third thiourea derivative, Fig. 2. The latter interactions link chains into a 2-D array with additional stabilisation afforded by C—H···π, Table 1, and π···π interactions. The latter occur between the benzene rings: ring centroid(C4–C9)···ring centroid(C12–C17)^i^ distance = 3.7821 (10) Å, with a dihedral angle between the least-squares plane through the rings of 2.25 (7)°; symmetry operation i: 1 - x, -y, 1 - z.

### Experimental

A mixture of thiocarbohydrazone (0.53 g, 0.005 mol) and 2-hydroxyacetophenone (1.36 g, 0.01 mol) in absolute ethanol was heated under reflux for 4–5 h in the presence of 1–2 drops of glacial acetic acid. The reaction mixture was allowed to cool to room temperature for 1 h. The light-yellow precipitate was filtered off and washed several times using absolute ethanol, and was purified by recrystallization from hot absolute ethanol and dried under vacuum over P_2_O_5_. Colourless prisms of (I) were obtained by slow evaporation of acetone solution at room temperature. Yield: 1.66 g, 88.0%. m.p. = 494–496 K. IR (KBr): ν_OH_ (3561–3391), ν_NH_ 3230, ν_C__═__N_ 1619, ν_N—N_ 963, ν_C__═__S_ 857 cm^-1^.

### Refinement

Carbon-bound H-atoms were placed in calculated positions (C—H 0.95–0.98 Å) and were included in the refinement in the riding model approximation with *U*_iso_(H) set to 1.2–1.5*U*_eq_(C). The O- and N-bound H-atoms were located in a difference Fourier map and were refined with O–H and N–H restraints of 0.84 (10) Å and 0.88 (10) Å, respectively, and with U_iso_(H) = 1.2U_eq_(N) and 1.5U_eq_(O).

### Crystal data

**Table:**

C_17_H_18_N_4_O_2_S·H_2_O	*F*(000) = 760
*M**_r_* = 360.43	*D*_x_ = 1.386 Mg m^−^^3^
Monoclinic, *P*2_1_/*n*	Mo *K*α radiation, λ = 0.71073 Å
Hall symbol: -P 2yn	Cell parameters from 4324 reflections
*a* = 15.8654 (3) Å	θ = 2.9–27.4°
*b* = 7.3938 (1) Å	µ = 0.21 mm^−^^1^
*c* = 16.3697 (3) Å	*T* = 100 K
β = 115.922 (1)°	Prism, colourless
*V* = 1727.06 (5) Å^3^	0.44 × 0.13 × 0.07 mm
*Z* = 4	

### Data collection

**Table:**

Bruker SMART APEXII CCD diffractometer	3968 independent reflections
Radiation source: sealed tube	3136 reflections with *I* > 2σ(*I*)
graphite	*R*_int_ = 0.033
φ and ω scans	θ_max_ = 27.5°, θ_min_ = 1.5°
Absorption correction: multi-scan (*SADABS*; Sheldrick, 1996)	*h* = −20→20
*T*_min_ = 0.905, *T*_max_ = 1	*k* = −9→8
15464 measured reflections	*l* = −21→21

### Refinement

**Table:**

Refinement on *F*^2^	Primary atom site location: structure-invariant direct methods
Least-squares matrix: full	Secondary atom site location: difference Fourier map
*R*[*F*^2^ > 2σ(*F*^2^)] = 0.038	Hydrogen site location: inferred from neighbouring sites
*wR*(*F*^2^) = 0.102	H atoms treated by a mixture of independent and constrained refinement
*S* = 1.03	*w* = 1/[σ^2^(*F*_o_^2^) + (0.0487*P*)^2^ + 0.7503*P*] where *P* = (*F*_o_^2^ + 2*F*_c_^2^)/3
3968 reflections	(Δ/σ)_max_ = 0.001
246 parameters	Δρ_max_ = 0.35 e Å^−^^3^
7 restraints	Δρ_min_ = −0.27 e Å^−^^3^

### Special details

**Table:**

Geometry. All s.u.'s (except the s.u. in the dihedral angle between two l.s. planes) are estimated using the full covariance matrix. The cell s.u.'s are taken into account individually in the estimation of s.u.'s in distances, angles and torsion angles; correlations between s.u.'s in cell parameters are only used when they are defined by crystal symmetry. An approximate (isotropic) treatment of cell s.u.'s is used for estimating s.u.'s involving l.s. planes.
Refinement. Refinement of *F*^2^ against ALL reflections. The weighted *R*-factor wR and goodness of fit *S* are based on *F*^2^, conventional *R*-factors *R* are based on *F*, with *F* set to zero for negative *F*^2^. The threshold expression of *F*^2^ > 2σ(*F*^2^) is used only for calculating *R*-factors(gt) etc. and is not relevant to the choice of reflections for refinement. *R*-factors based on *F*^2^ are statistically about twice as large as those based on *F*, and *R*- factors based on ALL data will be even larger.

### Fractional atomic coordinates and isotropic or equivalent isotropic displacement parameters (Å^2^)

**Table:**

	*x*	*y*	*z*	*U*_iso_*/*U*_eq_	
S1	0.65089 (3)	0.17338 (6)	0.56929 (3)	0.02002 (12)	
O1	0.65341 (7)	0.31213 (16)	0.77557 (8)	0.0213 (3)	
H1O	0.6224 (14)	0.300 (3)	0.7201 (15)	0.032*	
O2	0.70052 (8)	−0.01110 (17)	0.38996 (7)	0.0225 (3)	
H2O	0.6629 (14)	0.041 (3)	0.4040 (14)	0.034*	
O3	0.82009 (8)	0.13214 (18)	0.86971 (9)	0.0240 (3)	
H3O	0.7673 (15)	0.180 (3)	0.8392 (15)	0.036*	
H4O	0.8143 (14)	0.022 (3)	0.8646 (15)	0.036*	
N1	0.47863 (9)	0.29267 (19)	0.53363 (9)	0.0179 (3)	
H1N	0.4205 (13)	0.313 (2)	0.4972 (12)	0.022*	
N2	0.51214 (9)	0.33207 (18)	0.62387 (8)	0.0166 (3)	
N3	0.49527 (9)	0.19563 (19)	0.41174 (9)	0.0179 (3)	
H3N	0.4365 (13)	0.229 (3)	0.3827 (13)	0.021*	
N4	0.54462 (9)	0.12100 (18)	0.36967 (9)	0.0172 (3)	
C1	0.53819 (10)	0.2217 (2)	0.50290 (11)	0.0170 (3)	
C2	0.45776 (10)	0.4008 (2)	0.65560 (10)	0.0158 (3)	
C3	0.35589 (10)	0.4419 (2)	0.59818 (11)	0.0203 (3)	
H3A	0.3499	0.5603	0.5693	0.030*	
H3B	0.3227	0.4433	0.6365	0.030*	
H3C	0.3287	0.3488	0.5513	0.030*	
C4	0.50342 (10)	0.4407 (2)	0.75353 (10)	0.0163 (3)	
C5	0.59838 (11)	0.3942 (2)	0.80910 (11)	0.0175 (3)	
C6	0.63916 (11)	0.4332 (2)	0.90125 (11)	0.0206 (3)	
H6	0.7024	0.3997	0.9379	0.025*	
C7	0.58878 (11)	0.5202 (2)	0.94020 (11)	0.0225 (4)	
H7	0.6175	0.5468	1.0033	0.027*	
C8	0.49609 (11)	0.5689 (2)	0.88708 (11)	0.0223 (4)	
H8	0.4613	0.6295	0.9136	0.027*	
C9	0.45484 (11)	0.5287 (2)	0.79543 (11)	0.0191 (3)	
H9	0.3913	0.5619	0.7598	0.023*	
C10	0.50214 (11)	0.0843 (2)	0.28371 (10)	0.0172 (3)	
C11	0.39945 (11)	0.1186 (2)	0.22693 (11)	0.0235 (4)	
H11A	0.3647	0.0869	0.2618	0.035*	
H11B	0.3771	0.0445	0.1718	0.035*	
H11C	0.3896	0.2468	0.2101	0.035*	
C12	0.56124 (11)	0.0036 (2)	0.24484 (10)	0.0178 (3)	
C13	0.65610 (11)	−0.0429 (2)	0.29941 (11)	0.0187 (3)	
C14	0.70852 (11)	−0.1286 (2)	0.26121 (11)	0.0227 (4)	
H14	0.7713	−0.1634	0.2990	0.027*	
C15	0.67062 (12)	−0.1635 (2)	0.16938 (12)	0.0242 (4)	
H15	0.7073	−0.2212	0.1440	0.029*	
C16	0.57851 (12)	−0.1140 (2)	0.11390 (11)	0.0247 (4)	
H16	0.5523	−0.1360	0.0504	0.030*	
C17	0.52543 (12)	−0.0331 (2)	0.15140 (11)	0.0217 (4)	
H17	0.4624	−0.0010	0.1128	0.026*	

### Atomic displacement parameters (Å^2^)

**Table:**

	*U*^11^	*U*^22^	*U*^33^	*U*^12^	*U*^13^	*U*^23^
S1	0.01439 (18)	0.0259 (2)	0.01724 (19)	0.00368 (15)	0.00456 (14)	0.00086 (17)
O1	0.0168 (5)	0.0242 (6)	0.0187 (6)	0.0036 (5)	0.0039 (5)	−0.0026 (5)
O2	0.0162 (5)	0.0326 (7)	0.0167 (6)	0.0010 (5)	0.0054 (5)	−0.0034 (5)
O3	0.0144 (5)	0.0226 (7)	0.0283 (6)	0.0009 (5)	0.0031 (5)	−0.0023 (6)
N1	0.0135 (6)	0.0230 (8)	0.0150 (6)	0.0015 (5)	0.0042 (5)	−0.0006 (6)
N2	0.0165 (6)	0.0166 (7)	0.0148 (6)	−0.0005 (5)	0.0049 (5)	0.0007 (5)
N3	0.0142 (6)	0.0210 (7)	0.0162 (6)	0.0020 (5)	0.0046 (5)	−0.0002 (6)
N4	0.0170 (6)	0.0167 (7)	0.0176 (6)	−0.0003 (5)	0.0075 (5)	0.0003 (5)
C1	0.0168 (7)	0.0155 (8)	0.0180 (8)	−0.0017 (6)	0.0069 (6)	0.0014 (6)
C2	0.0152 (7)	0.0135 (8)	0.0181 (7)	−0.0021 (6)	0.0066 (6)	0.0014 (6)
C3	0.0165 (7)	0.0247 (9)	0.0182 (8)	−0.0006 (6)	0.0062 (6)	−0.0005 (7)
C4	0.0162 (7)	0.0139 (8)	0.0179 (7)	−0.0031 (6)	0.0066 (6)	0.0008 (6)
C5	0.0175 (7)	0.0130 (8)	0.0212 (8)	−0.0013 (6)	0.0078 (6)	0.0005 (6)
C6	0.0179 (7)	0.0185 (9)	0.0200 (8)	−0.0027 (6)	0.0032 (6)	0.0014 (7)
C7	0.0253 (8)	0.0226 (9)	0.0177 (8)	−0.0057 (7)	0.0075 (7)	−0.0023 (7)
C8	0.0247 (8)	0.0211 (9)	0.0243 (8)	−0.0022 (7)	0.0136 (7)	−0.0031 (7)
C9	0.0167 (7)	0.0194 (8)	0.0209 (8)	−0.0015 (6)	0.0081 (6)	0.0008 (7)
C10	0.0186 (7)	0.0132 (8)	0.0176 (7)	−0.0033 (6)	0.0058 (6)	0.0015 (6)
C11	0.0193 (8)	0.0236 (9)	0.0226 (8)	−0.0014 (6)	0.0046 (7)	0.0001 (7)
C12	0.0212 (8)	0.0136 (8)	0.0170 (7)	−0.0025 (6)	0.0069 (6)	0.0014 (6)
C13	0.0206 (7)	0.0181 (8)	0.0172 (7)	−0.0049 (6)	0.0080 (6)	0.0006 (6)
C14	0.0190 (8)	0.0249 (9)	0.0243 (8)	−0.0027 (6)	0.0096 (7)	0.0006 (7)
C15	0.0319 (9)	0.0202 (9)	0.0263 (9)	−0.0032 (7)	0.0181 (7)	−0.0024 (7)
C16	0.0360 (9)	0.0202 (9)	0.0169 (8)	−0.0018 (7)	0.0105 (7)	−0.0019 (7)
C17	0.0248 (8)	0.0168 (8)	0.0186 (8)	0.0003 (6)	0.0050 (7)	0.0008 (7)

### Geometric parameters (Å, °)

**Table:**

S1—C1	1.6754 (15)	C6—C7	1.380 (2)
O1—C5	1.3606 (19)	C6—H6	0.9500
O1—H1O	0.83 (2)	C7—C8	1.389 (2)
O2—C13	1.3550 (19)	C7—H7	0.9500
O2—H2O	0.82 (2)	C8—C9	1.382 (2)
O3—H3O	0.84 (2)	C8—H8	0.9500
O3—H4O	0.82 (2)	C9—H9	0.9500
N1—C1	1.355 (2)	C10—C12	1.470 (2)
N1—N2	1.3649 (18)	C10—C11	1.503 (2)
N1—H1N	0.865 (18)	C11—H11A	0.9800
N2—C2	1.290 (2)	C11—H11B	0.9800
N3—C1	1.356 (2)	C11—H11C	0.9800
N3—N4	1.3650 (18)	C12—C17	1.406 (2)
N3—H3N	0.876 (18)	C12—C13	1.416 (2)
N4—C10	1.2956 (19)	C13—C14	1.392 (2)
C2—C4	1.472 (2)	C14—C15	1.378 (2)
C2—C3	1.503 (2)	C14—H14	0.9500
C3—H3A	0.9800	C15—C16	1.391 (2)
C3—H3B	0.9800	C15—H15	0.9500
C3—H3C	0.9800	C16—C17	1.377 (2)
C4—C9	1.396 (2)	C16—H16	0.9500
C4—C5	1.420 (2)	C17—H17	0.9500
C5—C6	1.387 (2)		
			
C5—O1—H1O	108.2 (14)	C8—C7—H7	120.0
C13—O2—H2O	107.0 (14)	C9—C8—C7	119.54 (16)
H3O—O3—H4O	109 (2)	C9—C8—H8	120.2
C1—N1—N2	118.65 (13)	C7—C8—H8	120.2
C1—N1—H1N	121.2 (12)	C8—C9—C4	122.18 (14)
N2—N1—H1N	120.2 (12)	C8—C9—H9	118.9
C2—N2—N1	120.48 (12)	C4—C9—H9	118.9
C1—N3—N4	119.32 (13)	N4—C10—C12	115.31 (13)
C1—N3—H3N	117.4 (12)	N4—C10—C11	123.03 (15)
N4—N3—H3N	123.3 (12)	C12—C10—C11	121.65 (14)
C10—N4—N3	119.45 (13)	C10—C11—H11A	109.5
N1—C1—N3	111.55 (13)	C10—C11—H11B	109.5
N1—C1—S1	124.15 (12)	H11A—C11—H11B	109.5
N3—C1—S1	124.30 (12)	C10—C11—H11C	109.5
N2—C2—C4	114.84 (13)	H11A—C11—H11C	109.5
N2—C2—C3	123.59 (14)	H11B—C11—H11C	109.5
C4—C2—C3	121.55 (14)	C17—C12—C13	116.86 (15)
C2—C3—H3A	109.5	C17—C12—C10	121.22 (14)
C2—C3—H3B	109.5	C13—C12—C10	121.91 (14)
H3A—C3—H3B	109.5	O2—C13—C14	116.31 (14)
C2—C3—H3C	109.5	O2—C13—C12	123.28 (15)
H3A—C3—H3C	109.5	C14—C13—C12	120.40 (15)
H3B—C3—H3C	109.5	C15—C14—C13	120.93 (15)
C9—C4—C5	117.19 (14)	C15—C14—H14	119.5
C9—C4—C2	120.92 (13)	C13—C14—H14	119.5
C5—C4—C2	121.88 (14)	C14—C15—C16	119.74 (16)
O1—C5—C6	117.06 (14)	C14—C15—H15	120.1
O1—C5—C4	122.53 (14)	C16—C15—H15	120.1
C6—C5—C4	120.40 (15)	C17—C16—C15	119.75 (15)
C7—C6—C5	120.68 (15)	C17—C16—H16	120.1
C7—C6—H6	119.7	C15—C16—H16	120.1
C5—C6—H6	119.7	C16—C17—C12	122.27 (15)
C6—C7—C8	120.00 (15)	C16—C17—H17	118.9
C6—C7—H7	120.0	C12—C17—H17	118.9
			
C1—N1—N2—C2	179.58 (14)	C7—C8—C9—C4	−0.4 (3)
C1—N3—N4—C10	175.22 (15)	C5—C4—C9—C8	−0.4 (2)
N2—N1—C1—N3	−178.03 (13)	C2—C4—C9—C8	−179.56 (15)
N2—N1—C1—S1	1.7 (2)	N3—N4—C10—C12	−179.77 (13)
N4—N3—C1—N1	−178.53 (13)	N3—N4—C10—C11	−0.5 (2)
N4—N3—C1—S1	1.8 (2)	N4—C10—C12—C17	−176.26 (15)
N1—N2—C2—C4	−178.58 (13)	C11—C10—C12—C17	4.5 (2)
N1—N2—C2—C3	0.2 (2)	N4—C10—C12—C13	4.6 (2)
N2—C2—C4—C9	174.83 (15)	C11—C10—C12—C13	−174.62 (15)
C3—C2—C4—C9	−4.0 (2)	C17—C12—C13—O2	178.25 (15)
N2—C2—C4—C5	−4.3 (2)	C10—C12—C13—O2	−2.6 (2)
C3—C2—C4—C5	176.85 (15)	C17—C12—C13—C14	−2.9 (2)
C9—C4—C5—O1	−178.43 (14)	C10—C12—C13—C14	176.31 (15)
C2—C4—C5—O1	0.8 (2)	O2—C13—C14—C15	−178.53 (15)
C9—C4—C5—C6	1.0 (2)	C12—C13—C14—C15	2.5 (3)
C2—C4—C5—C6	−179.76 (15)	C13—C14—C15—C16	−0.5 (3)
O1—C5—C6—C7	178.47 (15)	C14—C15—C16—C17	−1.0 (3)
C4—C5—C6—C7	−1.0 (2)	C15—C16—C17—C12	0.6 (3)
C5—C6—C7—C8	0.3 (3)	C13—C12—C17—C16	1.4 (2)
C6—C7—C8—C9	0.4 (3)	C10—C12—C17—C16	−177.81 (15)

### Hydrogen-bond geometry (Å, °)

**Table:**

Cg1 is the centroid of the C4–C9 ring.

**Table:**

*D*—H···*A*	*D*—H	H···*A*	*D*···*A*	*D*—H···*A*
O1—H1O···N2	0.83 (2)	1.79 (2)	2.5191 (17)	147 (2)
O1—H1O···S1	0.83 (2)	2.86 (2)	3.5126 (13)	138 (2)
O2—H2O···N4	0.82 (2)	1.81 (2)	2.542 (2)	148 (2)
O2—H2O···S1	0.82 (2)	2.96 (2)	3.6220 (15)	139 (2)
O3—H3O···O1	0.84 (2)	1.91 (2)	2.7525 (18)	174 (2)
O3—H4O···S1^i^	0.82 (2)	2.76 (2)	3.5089 (14)	154 (2)
N1—H1N···O3^ii^	0.87 (2)	2.04 (2)	2.8169 (19)	150 (2)
N3—H3N···O3^ii^	0.88 (2)	2.04 (2)	2.854 (2)	154 (2)
C11—H11C···Cg1^iii^	0.98	2.61	3.4497 (17)	144

Symmetry codes:  (i) −*x*+3/2, *y*−1/2, −*z*+3/2; (ii) *x*−1/2, −*y*+1/2, *z*−1/2; (iii) −*x*+1, −*y*+1, −*z*+1.
